# Accurate approximation and MPI parallelization of spatial stochastic reaction-diffusion in STEPS

**DOI:** 10.1186/1471-2202-15-S1-P177

**Published:** 2014-07-21

**Authors:** Iain Hepburn, Weiliang Chen, Erik De Schutter

**Affiliations:** 1 Theoretical Neurobiology, University of Antwerp, 2610 Antwerpen, Belgium; 2 Computational Neuroscience Unit, Okinawa Institute of Science and Technology, Okinawa, 904-0411, Japan

## 

Spatial stochastic reaction-diffusion simulations have become an important component of molecular modeling in Computational Neuroscience, as shown in a growing number of recent studies including our previous work in which we show that stochastic effects, in particular stochastic calcium dynamics, contribute to Purkinje cell calcium burst variability [1]. However, the computational cost of exact, serial algorithms such as Gillespie’s direct method - as implemented in STEPS [2] - in which every reaction event and cross-subvolume diffusion event is simulated, impose severe restrictions on model complexity. Serial solutions limit the number of molecular species and their reaction channels that can be represented, as well as imposing restrictive upper-limits on the number of subvolumes in the discretized space. It is desirable to provide a solution that allows investigation of stochastic effects in larger systems, with complex meshes of many hundreds of thousands or millions of subvolumes representing complex neuronal morphology, in projects such as the Human Brain project [3], which is not possible with serial solutions. Therefore, parallel solutions for stochastic reaction-diffusion is vital for the future of this field.

There are common challenges of parallelization for reaction-diffusion simulators as well as some unique challenges for STEPS, in which space is discretized into irregular tetrahedrons and not cubes that are commonly used by other reaction-diffusion simulators. Exact solutions are not beneficial, where stochastic diffusion across geometry partitions result in frequent conflict between nodes, requiring regular costly rollbacks. Therefore it is essential that an approximation algorithm is applied. Such an approximation must minimise loss of accuracy whilst maximising performance gain.

Existing solutions such as the Gillespie-Multi-Particle method (GMP) [4,5] have been designed for cubic subvolumes, which have the advantage of regularity (yet restrict morphological accuracy [2]). We review such approaches from a theoretical standpoint and find that a systematic diffusion slowing error exists with naïve application to irregular subvolumes, which have local scaled diffusion coefficients and therefore localized expected diffusion times. We present a method that is tailored for irregular subvolumes, with a defined theoretical upper limit to the communication time-step. The method could, however, be generalised for different subvolume configurations where adaptable step-sizes could be beneficial. We further expand our method to allow multiple particle leaps, which for some systems this can greatly increase the minimum acceptable step-size and thus benefit performance whilst still ensuring acceptable accuracy. We demonstrate accuracy of our approximation by comparing to analytical solutions on a number of test cases.

Our method is implemented on an MPI framework, and we present performance gains under two scenarios- global application of the method, and application only at partition boundaries. We discuss the potential of this method for future large-scale parallel simulations in STEPS.
